# Lhx6 deficiency causes human embryonic palatal mesenchymal cell mitophagy dysfunction in cleft palate

**DOI:** 10.1186/s10020-024-00960-2

**Published:** 2024-10-22

**Authors:** Haotian Luo, Hio Cheng Ieong, Runze Li, Delan Huang, Danying Chen, Xin Chen, Yuqing Guo, Yangqiao Qing, Bingyan Guo, Ruoyu Li, Yungshan Teng, Wenfeng Li, Yang Cao, Chen Zhou, Weicai Wang

**Affiliations:** Hospital of Stomatology, Guanghua School of Stomatology, Guangdong Provincial Key Laboratory of Stomatology, Sun Yat-sen University, Guangzhou, Guangdong 510055 China

**Keywords:** Cleft palate, Retinoic acid, Embryonic palatal mesenchymal cells, Lhx6, PINK1, Mitophagy

## Abstract

**Background:**

Overconsumption of retinoic acid (RA) or its analogues/derivatives has been linked to severe craniomaxillofacial malformations, such as cleft palate and midface hypoplasia. It has been noted that RA disturbed the proliferation and migration of embryonic palatal mesenchymal (EPM) cells in these malformations, yet the exact mechanisms underlying these disruptions remained unclear.

**Methods:**

A model of retinoic acid (RA)-induced cleft palate in fetal mice was successfully established. Histological alterations in the palate were evaluated using Hematoxylin and Eosin (H&E) staining and RNA in situ hybridization (RNAscope). Cellular proliferation levels were quantified via the Cell Counting Kit-8 (CCK-8) assay and EdU incorporation assay, while cell migration capabilities were investigated using wound healing and Transwell assays. Mitochondrial functions were assessed through Mito-Tracker fluorescence, mitochondrial reactive oxygen species (ROS) measurement, ATP level quantification, and mitochondrial DNA (mtDNA) copy number analysis. Differential gene expression and associated signaling pathways were identified through bioinformatics analysis. Alterations in the transcriptional and translational levels of Lhx6 and genes associated with mitophagy were quantified using quantitative PCR (qPCR) and Western blot analysis, respectively. Mitochondrial morphology and the mitochondrial autophagosomes within cells were examined through transmission electron microscopy (TEM).

**Results:**

Abnormal palatal development in mice, along with impaired proliferation and migration of human embryonic palatal mesenchymal (HEPM) cells, was associated with RA affecting mitochondrial function and concomitant downregulation of Lhx6. Knockdown of Lhx6 in HEPM cells resulted in altered cell proliferation, migration, and mitochondrial function. Conversely, the aberrant mitochondrial function, proliferation, and migration observed in RA-induced HEPM cells were ameliorated by overexpression of Lhx6. Subsequent research demonstrated that Lhx6 ameliorated RA-induced dysfunction in HEPM cells by modulating PINK1/Parkin-mediated mitophagy, thereby activating the MAPK signaling pathways.

**Conclusion:**

Lhx6 is essential for mitochondrial homeostasis via tuning PINK1/Parkin-mediated mitophagy and MAPK signaling pathways. Downregulation of Lhx6 by RA transcriptionally disturbs the mitochondrial homeostasis, which in turn leads to the proliferation and migration defect in HEPM cells, ultimately causing the cleft palate.

**Graphical abstract:**

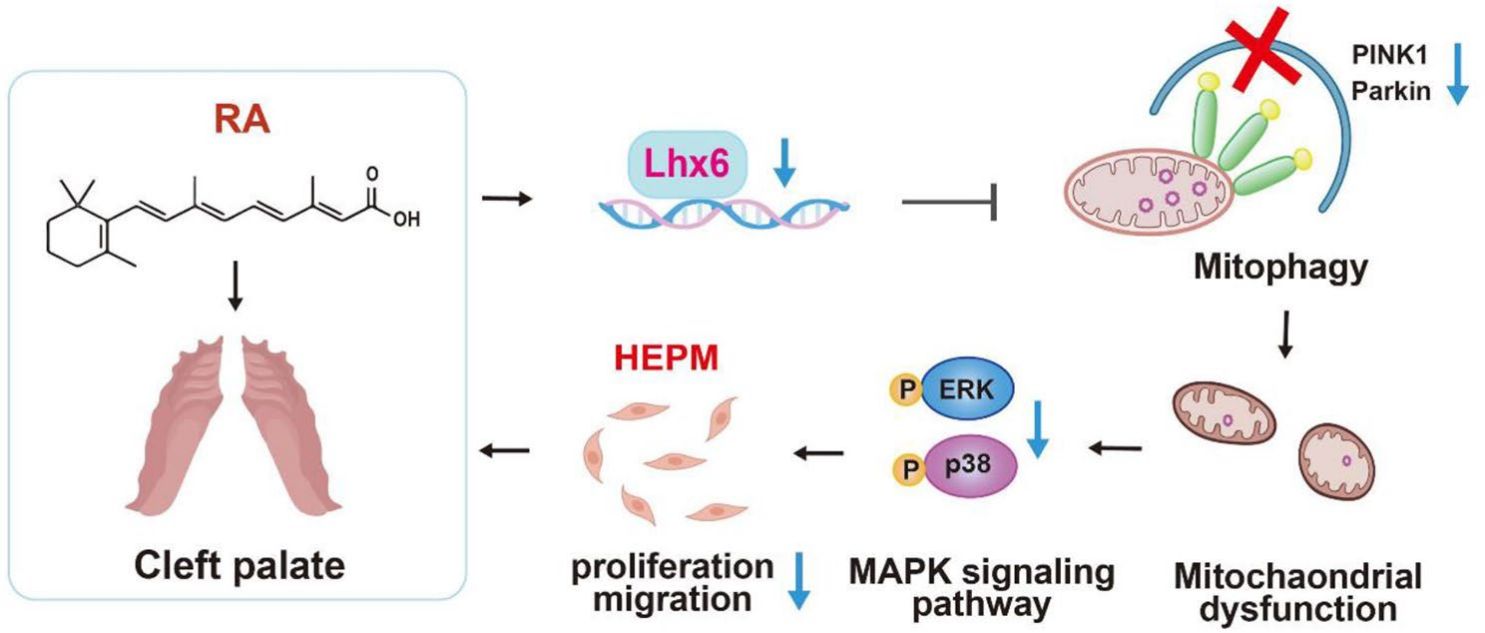

**Supplementary Information:**

The online version contains supplementary material available at 10.1186/s10020-024-00960-2.

## Introduction

Clefts of the lip and/or palate (CLP) are prevalent congenital malformations that affect approximately 1 in 700 individuals. CLP arises from inadequate growth or fusion of facial prominences during early embryonic development. Individuals with CLP experience challenges related to feeding, sucking, articulation, dental development, and mental well-being (Dixon et al. 2011), necessitating extensive therapeutic interventions. The multifaceted etiology of CLP involves a combination of genetic and environmental factors, such as exposure to chemicals, organic solvents, and tobacco smoke. Cleft palate is caused by abnormal function of mesenchymal stromal cells in palatal shelf and disruption of the palatal shelf uplift fusion caused by any factor in this process. Elucidating the biology and regulatory network of embryonic palatal mesenchymal (EPM) cells is of enormous importance.

Retinoic acid (RA), also known as all-trans retinoic acid (atRA) and vitamin A acid, is an intermediate metabolite of vitamin A with various therapeutic and cosmetic applications in dermatology. Its role in regulating epidermal mitosis and cell renewal makes it effective in treating conditions such as acne (Kakpovbia et al. 2024) and photoaging (Halai et al. 2024). Furthermore, RA influences tumor cell differentiation through vitamin A signaling, resulting in antiproliferative and proapoptotic effects (Mazziotta et al. 2024), also has a spectrum of plasticity for stem cell differentiation (Tierney et al. 2024), which is therapeutically important in cancer treatment. Exposure to RA, particularly during specific stages of development, can act as a teratogen by interfering with the proliferation and differentiation of embryonic mesenchymal cells, leading to abnormalities in craniomaxillofacial skeletal development (Yu et al. 2023). Overconsumption of RA during embryonic craniofacial development can lead to fetal craniomaxillofacial malformations such as cleft palate, midface hypoplasia, and premature closure of the middle cranial suture. But the precise mechanism behind this phenomenon remains unknown, highlighting the importance of elucidating it for the prevention of RA-induced developmental malformations.

Lhx6 and Lhx8, the LIM homeobox genes, encodes transcription factors that were proved in our previous studies to play crucial roles in craniofacial mesenchyme development and cell fate determination (Huang et al. 2021; Wang et al. 2019a; Zhou et al. 2015b). Lhx6/8 are important regulators in neurogenesis, odontogenesis, and palatal development in the context of craniomaxillofacial development (Zhou et al. 2015a), as well as, based on our study by optogenetics, have the great potential to enhance bone regeneration via promoting mesenchymal cell commitment (Huang et al. 2021). Knockdown of Lhx6 or Lhx6/8 in mouse result in multiple craniofacial defects, including cleft palate and missing molars (Denaxa et al. 2009). The aforementioned studies suggest that Lhx6 is a key player in embryonic development and the control of cell functions, making it a potential biomarker and therapeutic target for various diseases. Nevertheless, the precise role and underlying mechanism of Lhx6 in the pathogenesis of cleft palate remain incompletely understood.

Mitochondria are important organelles in eukaryotic cells responsible for producing adenosine triphosphate (ATP) through oxidative phosphorylation (OXPHOS), which is essential for cell metabolism and growth (Monzel et al. 2023). Under normal physiological circumstances, mitochondria safeguard normal cell proliferation and migration functions, promoting tissue homeostasis and development (Antico Arciuch et al. 2012). However, in pathological conditions, mitochondrial dysfunction can occur, leading to mitochondrial membrane damage, altered permeability, decreased membrane potential, disrupted oxidative phosphorylation, reduced ATP levels, and other cellular abnormalities (Li et al. 2024a). Ultimately, mitochondrial dysfunction results in impaired cellular functions including adhesion, migration, endocytosis, and proliferation (Li et al. 2024b). The impact of mitochondrial dysfunction on craniofacial developmental abnormalities cannot be overlooked. Research has demonstrated that aberrant mitochondrial responses in embryos can disrupt the development of the first pharyngeal arch, causing cleft palate and micrognathia (Lu et al. 2012). Moreover, mitochondrial dysfunction mediates inhibition of cranial neural crest cell (CNCC) proliferation and migration, ultimately leading to craniofacial defects in the embryo (Yan et al. 2020). The deficiency of the key component of the mitochondrial respiratory chain, Coenzyme Q10, results in the cardio-facio-cutaneous (CFC) syndrome, in which patients exhibit a distinctive facial phenotype characterized by bilateral temporal constrictions, a prominent forehead, and downward-sloping lid fissures (Aeby et al. 2007). Also, the destruction of mitochondria may affect the ameloblast differentiation as well as the cell proliferation and death of Hertwig’s epithelial root sheath, leading to abnormal tooth development and the development of odontogenic tumors (Kaneko et al. 1999; Zheng et al. 2024). These studies underscore the pivotal role of mitochondrial function in influencing embryonic developmental fate. Mitophagy, a specialized form of autophagy, plays a significant role in regulating mitochondrial quality and quantity, impacting early embryonic development, cell differentiation, inflammation, and apoptosis (Onishi et al. 2021). Disruption of mitophagy impaired mitochondrial functions, causing defective organelles and progressive accumulation of reactive oxygen species, resulting in cell and tissue damage (Palikaras et al. 2018), which highlights the potential of modulating mitophagy as a therapeutic strategy to enhance cell proliferation and migration. Developmental defects have been shown to be associated with the disruption of mitophagy homeostasis, as observed in conditions like Wolfram syndrome(Crouzier et al. 2022; Patergnani et al. 2024) and Barth syndrome(Russo et al. 2024; Wang et al. 2023). However, there is a notable absence of literature regarding the role of mitophagy in craniomaxillofacial malformations. This gap has motivated our investigation into whether the function of mitophagy is compromised in RA-induced cleft palate as well as HEPM cells.

This study elucidated the impact of RA on HEPM cell function and mitochondrial homeostasis, and examined the involvement of Lhx6 in these mechanisms. A fetal mice cleft palate model was established through RA gavage administration to pregnant mice. Through in vitro experiments and biological information analysis, it was determined that RA suppressed mitophagy by reducing Lhx6 expression, resulting in mitochondrial dysfunction, aberrant cell proliferation and migration, and ultimately leading to cleft palate formation. During this process, the PINK1/Parkin/MAPK signaling pathway emerged as a crucial component in the regulatory network. This research unequivocally elucidated a novel mechanism of developmental abnormalities induced by RA, identifying potential targets for further investigation in the pursuit of innovative approaches to cleft palate prevention or translational treatment.

## Materials and methods

### Animal experiments

Six to ten-week-old male and female mice were purchased from Sun Yat-Sen University. Based on previous studies (Dong et al. 2017; Zheng et al. 2023) and in accordance with the 3R principles (reduction, replacement, refinement), a sample size of 6 pregnant mice per group at various time points (E13.5, E14.5 and E18.5) was determined. Taking into account the treatments, different collection time points and the attrition rate in the present study, a total of 40 female C57BL/6 mice were utilized. Female C57BL/6 mice weighing 16–25 g were mated with male mice weighing 24–30 g. The appearance of the vaginal plug was designated as Embryonic Day 0.5 (E0.5), and female mice were considered pregnant if they exhibited a distended abdomen and a weight gain of 5 g within a 10-day period starting from the mating day. The pregnant mice were then randomly and equally divided into two groups: a control group and a retinoic acid treatment group. On day E12, experimental group was gavaged with 0.1 mg/g retinoic acid corn oil solution and control group was gavaged with the same dose of corn oil. Two groups were executed on day E13.5, E14.5 and E18.5 to obtain embryonic palates. All fetuses were used to count the incidence of cleft palate, and the representative embryonic palates from each pregnant mouse were selected for further staining.

### Hematoxylin and eosin(H&E) staining and morphologic analysis

The palate tissues were fixed in 4% polyformaldehyde for 24 h, dehydrated using a graded series of alcohol, and embedded in paraffin. The paraffin was cut into 6 μm thick sections. After dewaxing, all tissue sections were stained with hematoxylin and eosin(H&E). Then the sample sections were imaged with an Aperio AT2 slide scanner (Leica Biosystems, Germany).

### Cell culture

The HEPM cells were obtained from the American Type Culture Collection (ATCC, USA). Cells were cultured in Eagle’s Minimum Essential Medium (ATCC, USA) containing 10% fetal bovine serum (FBS, Gibco, USA), 1%GlutaMax (Gibco, USA) and 1% penicillin/streptomycin (Gibco, USA) at 37°C in a 5% CO_2_ cell culture incubator. Cells were passaged at 70%~80% confluence, and a medium change takes place every two days. Cells were treated with 9µmol/L retinoic acid (Solarbio, China) for 48 h.

### CCK-8 assay

Cell proliferation ability was evaluated by the Cell Counting Kit-8 (BIOFIVEN, China). Cells were seeded in 96-well plates (3 × 10^3^cells/well) and cultured at 37°C.From day 0 to day 5, 10µL of CCK-8 reagent was added to each well, and incubated at 37°C in the dark for 2 h. Finally, the absorbance value at 450 nm was measured by a microplate spectrophotometer (BioTek, UK).

### EdU proliferation test

Cells were seeded in confocal dish overnight. A EdU incorporation assay (Beyotime, China) in accordance with the manufacturer’s instructions was used to determine cell proliferation. Cells were incubated with EdU at 37°C for 2 h, fixed with 4% polyformaldehyde at room temperature for 15 min, permeabilized with 0.3% Triton X-100 (MYMbio, China) and stained with Click Reaction Buffer at room temperature in the dark for 30 min. Finally, the nuclei were stained with Hoechst33342 (Beyotime, China) for 10 min. The staining was observed using a laser scanning confocal microscope (Olympus, Japan).

### Wound healing assay

The migration ability of cells was evaluated using wound healing assay. After a straight line was drawn across the bottom of a 6-well plate to locate the wounds, the cells were seeded in the plate at 5 × 10^5^ cells/well and incubated overnight at 37°C. A scratch wound was created with a 200µL pipette tip. The detached cells were then removed with 1×PBS (Biosharp, China). 2mL of serum-free α-MEM basal medium was added to each well. Finally, the cell migration distance at 0 h, 6 h and 12 h were observed and photographed by a cell imaging system (Invitrogen EVOS FL, Thermo Fisher Scientific, USA) and analyzed using ImageJ software (NIH, USA).

### Transwell migration assay

The Transwell cell culture inset of an 8.0 μm pore size was added into a 24-well plate to form the upper and lower chamber. 200µL serum-free medium with 2 × 10^4^ cells were loaded to the upper chamber, and 600µL medium containing 10% FBS was added to the lower chamber. After culturing for 24 h, cells in the upper chamber were wiped by cotton swabs. Cells migrated to the lower chamber were fixed with 4% polyformaldehyde at room temperature for 30 min and stained by 0.1% crystal violet (Solarbio, China) for 15 min. Migrated cells were counted under the inverted fluorescence microscope (Zeiss, Germany).

### Mito-Tracker fluorescence assay

Cells were incubated with Mito-Tracker Deep Red (Yeasen, China) in accordance with the manufacturer’s instructions at 37°C in the dark for 30 min and Hoechst33342 (Beyotime, China) for 10 min. Mitochondrial fluorescence was observed by a laser scanning confocal microscope (Olympus, Japan).

### Determination of ATP content

The cells were seeded in a 6-well plate. ATP contents were determined by lysing the cells and using an ATP Assay Kit (Beyotime, China) in accordance with the manufacturer’s instructions. Relative Light Unit (RLU) value was measured on a multifunctional microplate reader (BioTek, UK) and mitochondrial ATP content was calculated according to the standard curve.

### Mitochondrial DNA (mtDNA) copy number detection

Total DNA was extracted from the cell samples with TIANamp Genomic DNA Kit (TIANGEN BIOTECH, China) in accordance with the manufacturer’s instructions. Relative quantification of mitochondrial DNA was determined by quantitative real-time PCR using primers for MT ND1 (Generay Biotechnology, China) and β-actin (Generay Biotechnology, China). The relative mtDNA copy number was calculated using the 2^−△△CT^ method.

### Mitochondrial ROS detection

After cells were seeded in confocal dish overnight, cells were incubated in MitoSOX Red Mitochondrial Superoxide Indicator (Yeasen, China) at 37°C in the dark for 10 min and washed three times in PBS. The nuclei were stained with Hoechst33342 (Beyotime, China) for 10 min. Mitochondrial ROS levels were determined by a laser scanning confocal microscope (Olympus, Japan).

### Mitochondrial membrane potential assessment

Mitochondrial membrane potential was assessed using the JC-1 detection kit (JC-1 aggregate: red fluorescence; JC-1 monomer: green fluorescence; Beyotime, China). Cells were seeded in confocal dish overnight. According to the manufacturer’s instructions, JC-1 staining solution was loaded to the cells in serum-free medium at 37°C in the dark for 20 min. Finally, the nuclei were stained with Hoechst33342 (Beyotime, China) for 10 min and washed with PBS (Biosharp, China). Images were observed by a laser scanning confocal microscope (Olympus, Japan) and fluorescence intensity was analyzed using ImageJ software (NIH, USA).

### RNA in situ hybridization (RNAscope)

To localize Lhx6 expression in the palate tissue sections, a RNAscope^®^ 2.5HD Assay Kit (Bio-techne, USA) was used in accordance with the manufacturer’s instructions. After dewaxing, hydrogen peroxide blocking, and heat-mediated antigen retrieval using a steamer, Protease Plus was applied. After the hybridization probe and Hybridize Amp1-6 were added dropwise in sequence, diaminobenzidine (DAB, Servicebio, China) was used for visualization of Lhx6, and the slides were then counterstained and mounted.

### mRNA isolation and quantitative real-time PCR

mRNA was extracted from the cell samples using the RNA-Quick Purification Kit (Esun, China) and reverse transcribed into cDNA using the PrimeScript RT Master Mix (Takara, Japan) in accordance with the manufacturer’s instructions. Quantitative real-time PCR was performed using the AceQ^®^ Universal SYBR qPCR Master Mix (Vazyme, China). GAPDH was used as the internal reference gene for the total RNA. The specific primer sequences can be shown in Table S1. The relative expression of targeted genes was calculated using the 2^−△△CT^ method.

### Western blots

Total protein was extracted from cell samples using RIPA lysis buffer (CoWin Biosciences, China) with Protease Inhibitor Cocktail (Transgene, China) and phosphatase inhibitors (Servicebio, China). The protein concentration was measured by a BCA Protein Assay Kit (CoWin Biosciences, China). The proteins from samples were separated by SDS-PAGE (Genscript, China) electrophoresis for 1 h and transferred to polyvinylidene fluoride (PVDF) membranes (Millipore, USA). After blocking the membranes with 5% bovine serum albumin (Biofroxx, Germany) at room temperature for 1 h, the membranes were incubated at 4°C overnight with primary antibodies: polyclonal rabbit anti-Lhx6 (1:500, Bioss, China), monoclonal rabbit anti-LC3 (1:1000, CST, USA), polyclonal rabbit anti-Beclin1 (1:500, Boster, China), polyclonal rabbit anti-p62 (1:500, Servicebio, China), monoclonal rabbit anti-ATG5 (1:1000, CST, USA), polyclonal rabbit anti-PINK1 (1:500, Affinity Biosciences, China), polyclonal rabbit anti-Parkin (1:500, Affinity Biosciences, China), polyclonal rabbit anti-TOMM20 (1:500, Affinity Biosciences, China), monoclonal rabbit anti-Phospho-p38 MAPK (1:1000, CST, USA), monoclonal rabbit anti-p38 MAPK (1:1000, CST, USA), monoclonal rabbit anti-Phospho-p44/42 MAPK (ERK1/2) (1:1000, CST, USA), monoclonal rabbit anti-p44/42 MAPK (ERK1/2) (1:1000, CST, USA), polyclonal rabbit anti-β-actin (1:1000, Affinity Biosciences, China). The membranes were flushed with TBST for three times and incubated with the relevant secondary anti-rabbit antibody conjunct HRP agent (1:10000, CST, USA) at room temperature for 1 h. The band signals were measured using the ECL western blotting substrate kit (Millipore, USA) and analyzed using ImageJ software (NIH, USA).

### Mitochondrial and cytoplasmic fractionation

Mitochondria were extracted from the cells using the Cell Mitochondria Isolation Kit (Beyotime, China) in accordance with the manufacturer’s instructions. After separation of mitochondria by differential centrifugation, the supernatant was collected as the cytoplasmic fraction. Mitochondria and cytoplasmic were for further experiments.

### Transcriptome and differential gene expression

HEPM cell samples from the control groups (shCtrl, ovCtrl) and experimental groups (shLhx6, ovLhx6) were prepared for RNA library construction and whole-transcriptome sequencing by BGI Genomics Co, Ltd.,(China). To determine the differentially expressed genes between two different groups, the threshold for DEGs was *P* value < 0.05 and log2(fold change) > 1 or log2(fold change) < -1 were used. Genes between two different groups were analyzed for the overlaps using the Venn analysis. The data were analyzed for functional enrichment in order to better support functionality of the intend objectives. The signaling pathways between DEGs were revealed using Kyoto Encyclopedia of Genes and Genomes (KEGG) Pathway enrichment analyses, and MAPK pathway associated with DEGs were used for heat map visualization.

### Transmission electron microscope (TEM) assay

After digesting cells with TrypLE (Gibco, USA), the cells were centrifuged to form cell pellets and the culture medium was removed. The cell pellets were fixed with an electron microscope fixative (Servicebio, China) at room temperature in the dark for 1 ~ 2 h, and transferred to 4°C overnight. Subsequently, the cell pellets were dehydrated in an ethanol gradient and 100% acetone, and embedded in resin. Thin sections were then cut using an ultrathin microtome and stained with 3% uranyl acetate and lead citrate. The ultrastructure was observed by a transmission electron microscope (Hitachi, Japan).

### Cell transfection

For the overexpression of *LHX6* in HEPM cells, an amplified gene fragment encoding human *LHX6*(Gene ID:26468) was cloned into a lentiviral expression vector (Genepharma, China). HEPM cells were transfected with the *LHX6* or the negative control lentivirus at a Multiplicity of Infection (MOI) with 50:1 for 24 h. Subsequently, the transfected cells were chosen using lentivirus free medium containing 2 µg/mL Puromycin dihydrochloride (Puro, APExBIO, USA), and were collected for further experiments.

For the knockdown of Lhx6 in HEPM cells, a shRNA was cloned in a lentiviral vector (Genepharma, China). HEPM cells were transfected with the lentiviral mediated shRNA or negative control at an MOI with 50:1 for 24 h and chosen using medium containing 2 µg/mL Puro (APExBIO, USA) for further experiment.

For the knockdown of *PINK1* in HEPM cells, specific small interfering RNA (siRNA) targeting the Homo sapiens *PINK1* (Gene ID:65018) gene was transfected into HEPM cells using siRNA-mate plus transfection reagent kit (Genepharma, China) in accordance with the manufacturer’s instructions. The siRNA sense strand sequence:5’GCUGGAGGAGUAUCUGAUATT3’ and 5’GAAGCCACCAUGCCUACAUTT3’, the antisense strand sequence:5’UAUCAGAUACUCCUCCAGCTT3’ and 5’AUGUAGGCAUGGUGGCUUCTT3’. The transfected cells were collected for further experiments.

### Statistical analysis

All experimental values were derived from a minimum of three independent biological replicates. All data are presented as mean ± standard deviation (SD) with at least three independent experiments, and were analyzed using GraphPad Prism 9.0 software (USA). Comparisons between two groups were performed using the unpaired Student’s two-tailed *t*-test, and comparisons between more than two groups were performed using one-way analysis of variance (ANOVA). Differences between groups are considered to be non-significant (^NS^*P****>*** 0.05) or significant (^*^P < 0.05, ^**^P < 0.01, ^***^P < 0.001 vs. Ctrl).

## Results

### RA hindered the normal development of palatal shelves in mice and inhibited the proliferation and migration of HEPM cells

**Fig. 1:**
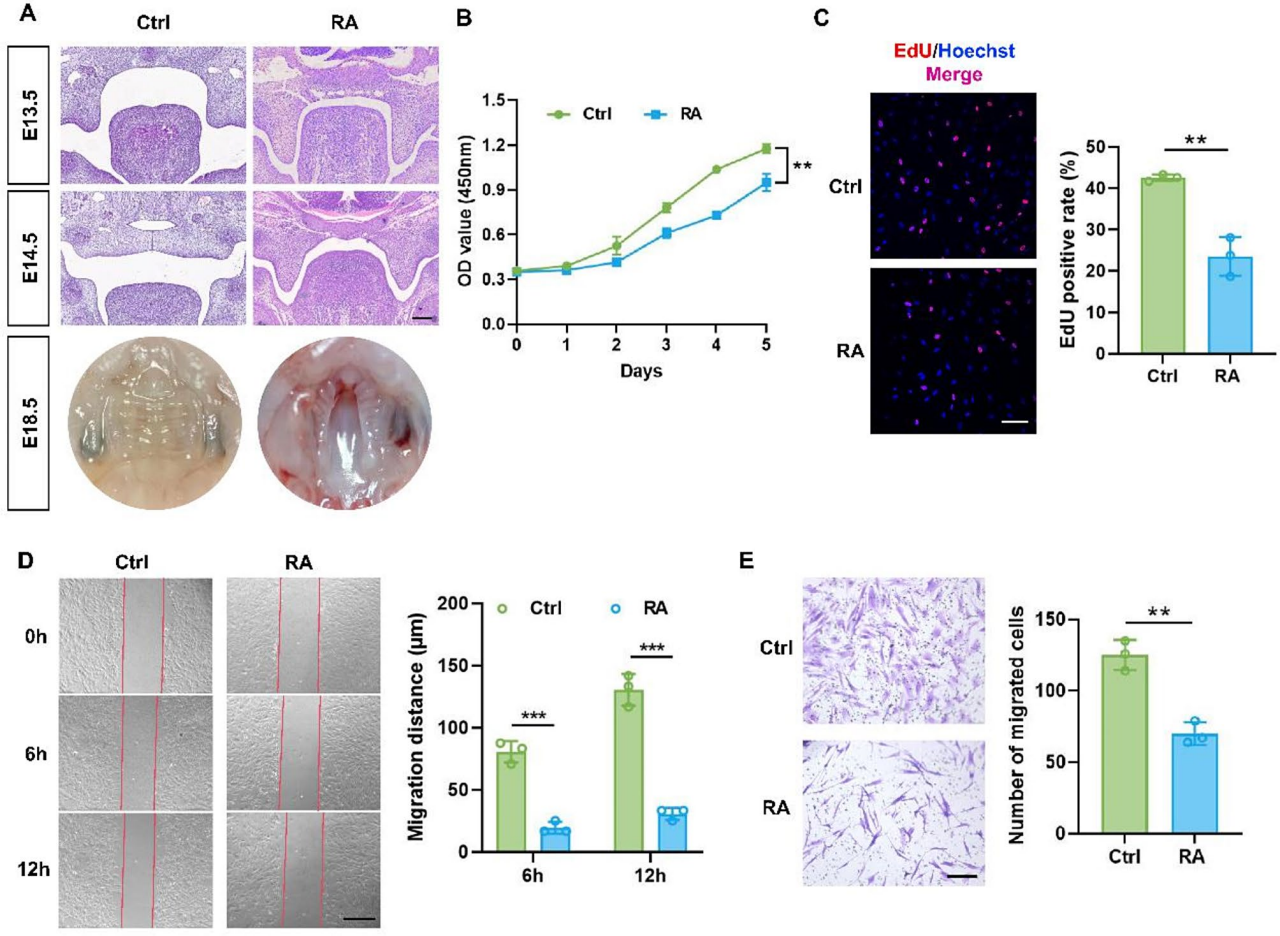
RA hindered the normal development of palatal processes and inhibited the proliferation and migration of HEPM cells. (**A**) The H&E staining of the palatal processes on both sides of the Ctrl group and the RA group. (*n* = 6 mice each group) Scale bar = 100 μm. (**B**) CCK-8 assay showed that RA inhibited the cell viability of HEPM cells. (**C**) Representative images and quantitative analysis of EdU staining (Red) of HEPM cells in the two groups. Nuclear DNA was counterstained with Hoechst. Scale bar = 100 μm. (**D**) The scratch assay and quantitative analysis of HEPM cells in the Ctrl group and the RA group. Scale bar = 500 μm. (**E**) Transwell assay and statistical analysis to evaluate HEPM cells migration. Scale bar = 200 μm. Data in the bar diagrams are represented as mean ± SD. N = at least 3 biological replicates. Circles correspond to each tested individual. **P < 0.01, ***P < 0.001

To verify the effects of RA on embryonic development, the pregnant mice were gavaged with 0.1 mg/g RA corn oil suspension for a fetal cleft palate model on day E12. The samples of embryos were obtained on days E13.5, E14.5 and E18.5 to observe the development of the palate, and the incidence of cleft palate was 85.4% (88/103). According to HE staining, embryos in the RA group were unable to lift up both sides of their palatal shelves on day E13.5, in contrast to those in the Ctrl group. By day E14.5, the palatal shelves of the Ctrl group had fused, whereas those of the RA group did not fuse and with no elevation. As of day E18.5, the palate of the Ctrl group had completed development, while the RA group had a cleft palate and the nasal cavity was perforated connecting with oral cavity (Fig. 1A).

Next, HEPM cells were used for in vitro experiments. After 48 h of treatment with 9µmol/L RA, the proliferation and migration of HEPM cells were altered. CCK-8 assay revealed that cells in the RA group proliferated at a slower rate than Ctrl (Fig. 1B). EdU staining indicated a significant reduction in positive cells in the RA group. In comparison with the Ctrl group, HEPM cells treated with RA showed decreased viability and inhibition of proliferation (Fig. 1C). Additionally, cell scratch assay (Fig. 1D) and Transwell migration assay (Fig. 1E) showed similar results, indicating that RA reduced HEPM cell migration ability. According to these findings, RA prevent palatal processes from lifting and merging. After treated with RA, proliferation and migration of HEPM cell were also inhibited.

### RA induced the mitochondrial dysfunctions of HEPM cells

**Fig. 2:**
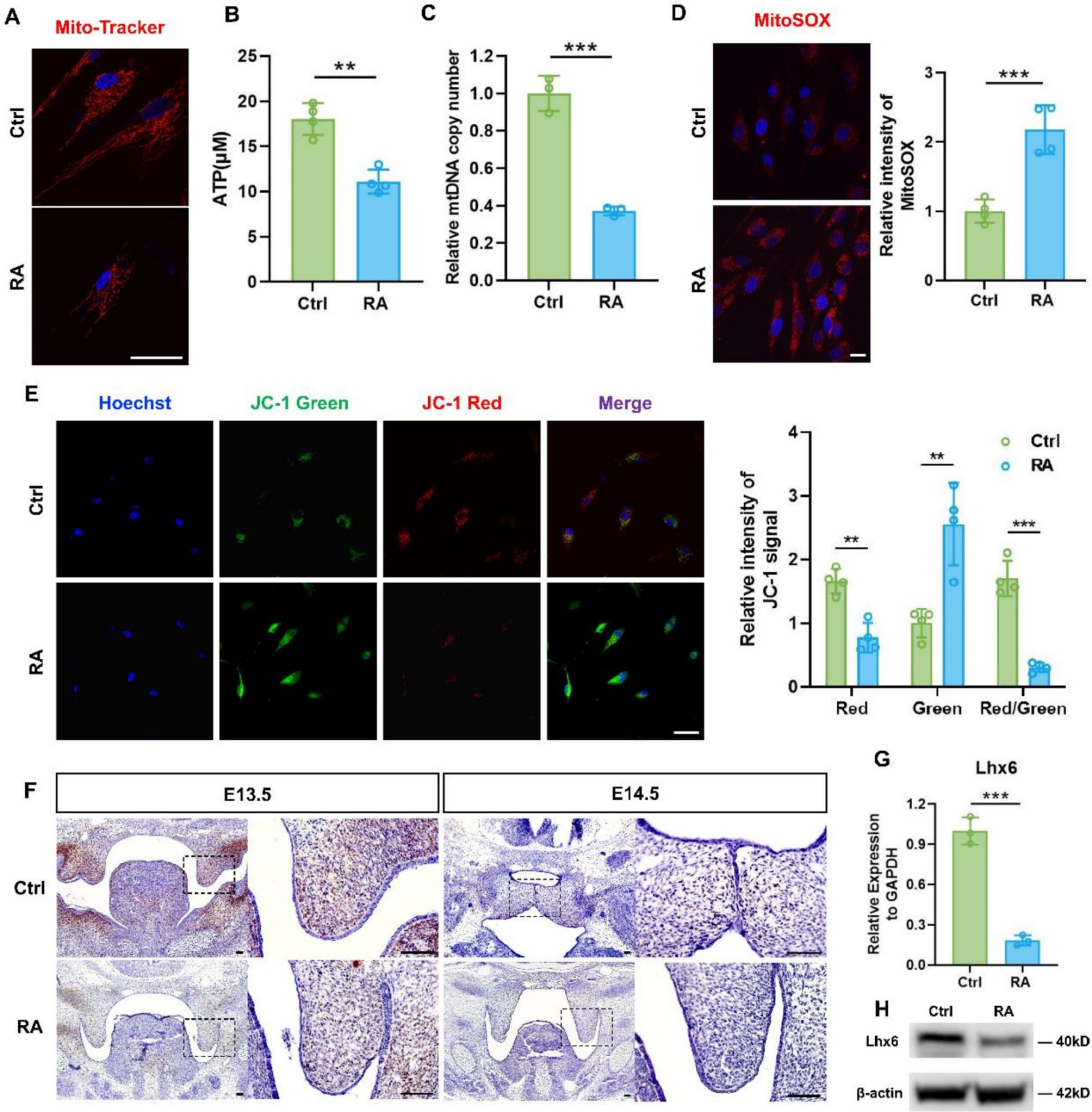
RA affected the mitochondrial functions and Lhx6 expression of HEPM cells. (**A**) Mito-Tracker Red fluorescence staining to observe the morphology and number of mitochondria in the two groups. Nuclear DNA was counterstained with Hoechst. Scale bar = 20 μm. The concentration of ATP (**B**) and the mtDNA copy number (**C**) were measured after 48 h of RA treatment. (**D**) Representative images of MitoSOX staining in HEPM cells and quantification of the two groups. Nuclear DNA was counterstained with Hoechst. Scale bar = 20 μm. (**E**) Mitochondrial membrane potential was measured by the fluorescence and quantitative analysis of JC-1. Nuclear DNA was counterstained with Hoechst. Scale bar = 50 μm. (**F**) RNAscope staining images of Lhx6 in palatal processes from the two groups are shown. Scale bar = 50 μm. (**G**) qPCR analysis of Lhx6 gene expression in the two groups. (**H**) The protein expression of Lhx6 was evaluated by western blots. Data in the bar diagrams are represented as mean ± SD. N = at least 3 biological replicates. Circles correspond to each tested individual. **P < 0.01, ***P < 0.001

As important energy-donating organelles, mitochondria play a crucial role in cell proliferation and migration activities. Here, we explored the changes in mitochondrial function in HEPM cells after RA treatment. Mito-Tracker probes were used for observing the changes in mitochondrial morphology and number, and Mito-Tracker RED staining revealed that, compared with the Ctrl group, rod-shaped mitochondria were reduced, dot-shaped mitochondria increased, and the fluorescence was weaker in the RA group (Fig. 2A), indicating that mitochondria were in poor condition and became fewer in number. It was detected that ATP levels (Fig. 2B) and mitochondrial copy numbers (Fig. 2C) decreased in the RA group, indicating a decrease in mitochondrial capacity. Mitochondria are also a major source of reactive oxygen species (ROS). To detect mtROS with MitoSOX fluorescent probes, we found enhanced fluorescence and higher mtROS in RA-induced HEPM cells (Fig. 2D). It is a sign of mitochondrial damage when the membrane potential of the mitochondria decreases. As shown in Fig. 2E, a decrease in mitochondrial membrane potential was evident in the JC-1 results after treated with RA, indicating damaged mitochondria. Based on the above results, RA may lead to abnormal mitochondrial morphology, dysfunction, and elevated mtROS levels.

### The expression of Lhx6 decreased in RA-treated HEPM cells

In previous studies, our group had demonstrated that the homeobox gene Lhx6/8 regulates craniofacial development in an important manner(Huang et al. 2021; Zhou et al. 2015a). In this study, we aimed to further investigate the relationship of RA and Lhx6 and their effect on the palatal protrusion of embryonic mice and HEPM cell function. According to RNAscope staining results, in normal condition, Lhx6 expression exhibited a dynamic manner throughout embryonic development, and it decreased on day E14.5 from day E13.5. RA resulted in lower expression of Lhx6 on days E13.5 and E14.5 than the Ctrl group (Fig. 2F). In vitro experiments confirmed that RA had a significant impact on both transcriptional (Fig. 2G) and translational (Fig. 2H) levels of the Lhx6 gene in HEPM cells. Collectively, RA downregulated the expression of Lhx6 both in vivo and in vitro.

### Knockdown of Lhx6 in HEPM cells impaired proliferation and migration, as well as mitochondrial function

**Fig. 3:**
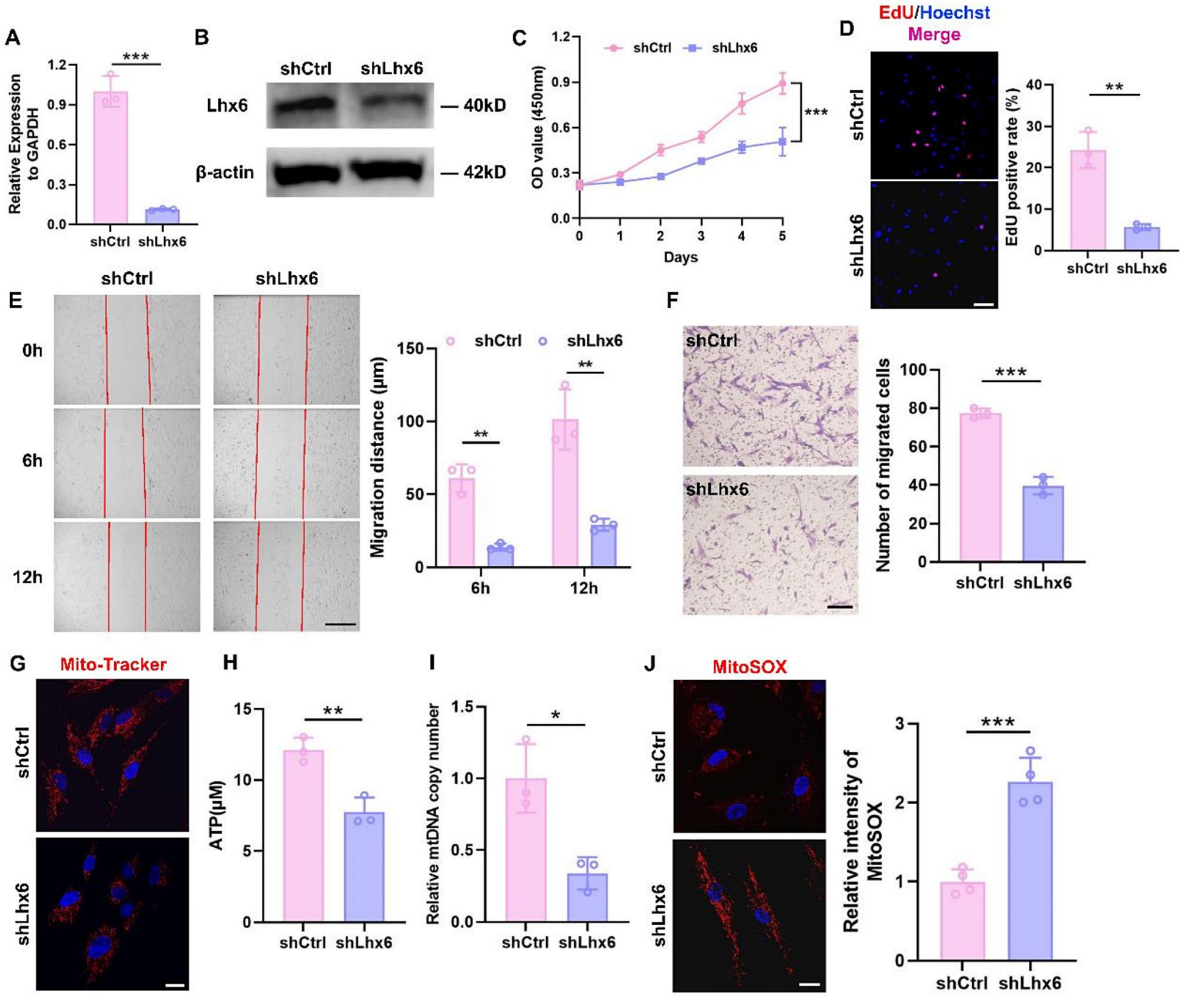
HEPM cells with knockdown of Lhx6 showed impaired proliferation and migration, as well as mitochondrial dysfunction. (**A**) qPCR analysis to confirm the efficiency of Lhx6 knockdown. (**B**) The protein expression of Lhx6 was evaluated by western blots. (**C**) The CCK-8 assay showed that Lhx6 knockdown inhibited HEPM cell viability. (**D**) Representative images and quantitative analysis of EdU staining (Red) of HEPM cells in the two groups. Nuclear DNA was counterstained with Hoechst. Scale bar = 100 μm. (**E**) The scratch assay and quantitative analysis of HEPM cells in shCtrl group and shLhx6 group. Scale bar = 500 μm. (**F**) Transwell assay and statistical analysis to evaluate cell migration. Scale bar = 200 μm. (**G**) Mito-Tracker Red fluorescence staining to observe the mitochondrial morphology and number in the two groups. Nuclear DNA was counterstained with Hoechst. Scale bar = 20 μm. The concentration of ATP (**H**) and the mtDNA copy number (**I**) were measured after knockdown of Lhx6. (**J**) Representative images of MitoSOX staining in HEPM cells and statistical analysis of the two groups. Nuclear DNA was counterstained with Hoechst. Scale bar = 20 μm. Data in the bar diagrams are represented as mean ± SD. N = at least 3 biological replicates. Circles correspond to each tested individual. *P < 0.05, **P < 0.01, ***P < 0.001

To investigate whether RA regulates mitochondrial function and cell proliferation and migration ability via Lhx6, HEPM cells were engineered with knockdown of Lhx6 and the efficiency was confirmed at transcriptional (Fig. 3A) and translational levels (Fig. 3B). HEPM cells proliferated less after Lhx6 knockdown, as indicated by the decreased proliferation rate (Fig. 3C) and the reduced number of EdU-positive cells (Fig. 3D). As to migration ability after knocking down Lhx6, we found similar results with RA-treated experiment in cell scratch assay and Transwell migration assay, where cells migrated less distantly (Fig. 3E), and fewer cells crossed the Transwell (Fig. 3F). Furthermore, knockdown of Lhx6 induced mitochondrial dysfunction, as evidenced by the reduced number of mitochondria with a predominance of dot-shaped mitochondria (Fig. 3G), decreased ATP levels (Fig. 3H) and mtDNA copy number (Fig. 3I), as well as elevated mtROS levels (Fig. 3J). Interestingly, we also found that Lhx6 downregulated the expression levels of ICT1 and MTRFR, which are two important mitoribosome-associated quality control (mtRQC) related factors (Fig. S1). Therefore, Lhx6 has been shown to be an important transcription factor in the regulation of cellular and mitochondrial functions in HEPM cells.

### Lhx6 overexpression restored the abnormal mitochondrial function, proliferation and migration in RA-induced HEPM cells

**Fig. 4:**
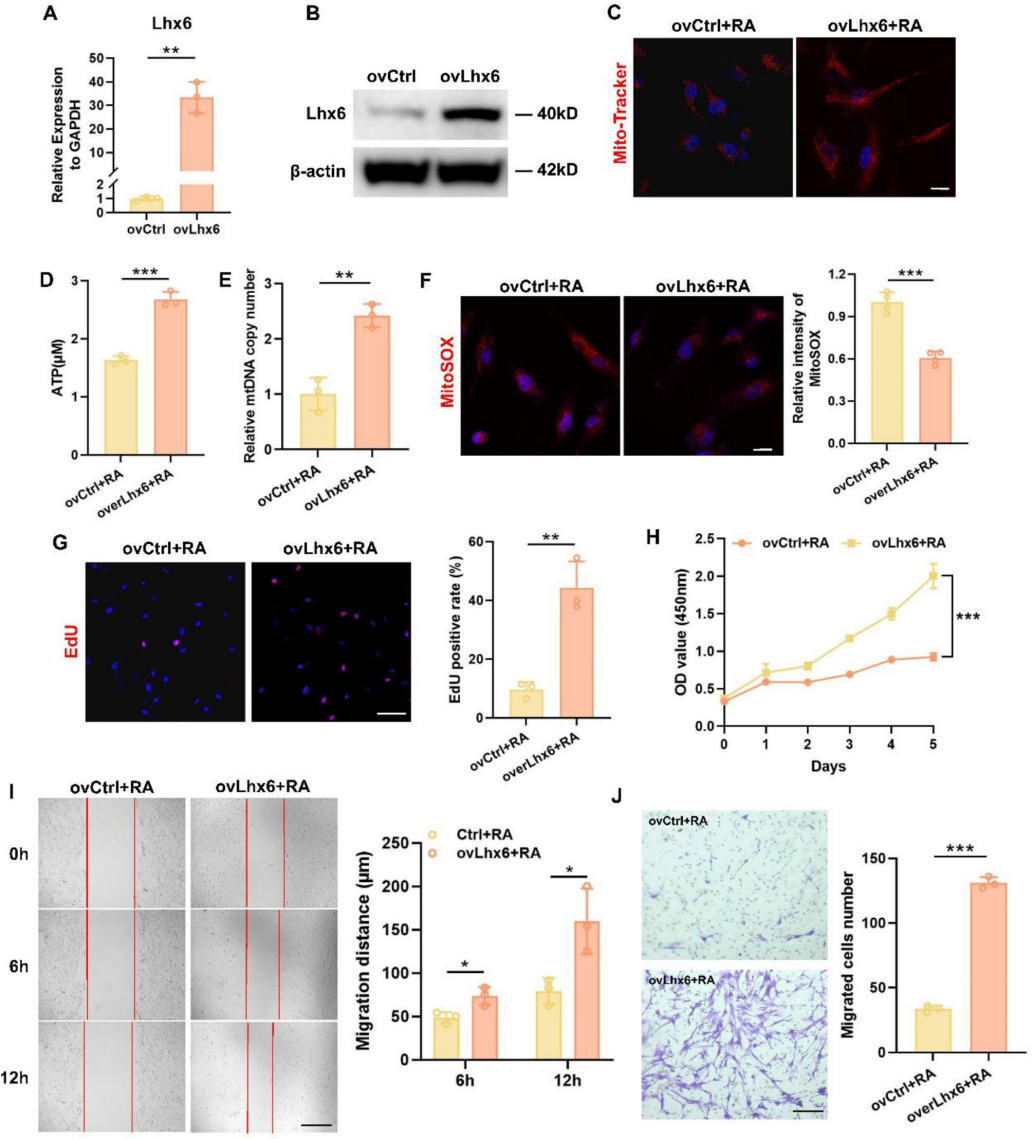
Lhx6 overexpression restored the abnormal mitochondrial function, proliferation and migration function in RA-induced HEPM cells. (**A**) qPCR analysis to confirm the efficiency of Lhx6 transfection. (**B**) The protein expression of Lhx6 was evaluated by western blots. (**C**) Mito-Tracker Red fluorescence staining to observe the morphology and number of mitochondria in the two groups. Nuclear DNA was counterstained with Hoechst. Scale bar = 20 μm. The concentration of ATP (**D**) and the mtDNA copy number (**E**) were measured in the two groups. (**F**) Representative images of MitoSOX staining in HEPM cells and quantification of the ovCtrl + RA and the ovLhx6 + RA groups. Nuclear DNA was counterstained with Hoechst. Scale bar = 20 μm. (**G**) Representative images and quantitative analysis of EdU staining (Red) of HEPM cells in the two groups. Nuclear DNA was counterstained with Hoechst. Scale bar = 100 μm. (**H**) CCK-8 assay showed that knockdown of Lhx6 inhibited cell viability of HEPM cells. (**I**) The scratch assay and quantitative analysis of HEPM cells in two groups. Scale bar = 500 μm. (**J**) Transwell assay and statistical analysis to evaluate cell migration. Scale bar = 200 μm. Data in the bar diagrams are represented as mean ± SD. N = at least 3 biological replicates. Circles correspond to each tested individual. *P < 0.05, **P < 0.01, ***P < 0.001

To further clarify the role of Lhx6 in the process of RA mediated mitochondrial and cellular functions, we constructed HEPM cells stably overexpressing Lhx6 and verified the expression level of Lhx6 by qPCR (Fig. 4A) and Western blots (Fig. 4B). A change in mitochondrial and cellular functions was observed after the Ctrl and overexpression Lhx6 (ovLhx6) groups were treated with RA. It is shown in Fig. 4C that the fluorescence of the Mito-Tracker Red fluorescent probe was significantly enhanced in the ovLhx6 + RA group, suggesting an increase in mitochondria. The morphology of mitochondria was rod-shaped, indicating that they were in a healthy stage in the ovLhx6 + RA group. The ATP level (Fig. 4D) and mtDNA copy number (Fig. 4E) increased, implying elevated mitochondrial production capacity. Using the MitoSOX fluorescent probe, mtROS levels were detected in mitochondria (Fig. 4F). In ovLhx6 + RA group, the fluorescence levels were decreased compared with ovCtrl + RA group, indicating decreased mtROS levels. We also found that Lhx6 restored the expression levels of ICT1 and MTRFR in RA-induced HEPM cells (Fig. S2).

In terms of cellular functions, according to the result of EdU staining (Fig. 4G) and the CCK-8 assay (Fig. 4H), cells in the ovLhx6 + RA group were more likely to be in a proliferative phase, had better cell viability and a higher proliferative capacity than the ovCtrl+RA group. A cell scratch assay (Fig. 4I) and a Transwell migration assay (Fig. 4J) elucidated that the cells in the ovLhx6 + RA group presented an increased migration distance, a sharp increase in the number of cells migrating through the chambers, and thus a significant improvement in cell migration ability.

The above results confirmed that Lhx6 reversed RA-induced mitochondrial dysfunction, restored the mitochondrial morphology and number, promoted the mitochondrial energy production and reduce its mtROS level, as well as enhanced the proliferation and migration of HEPM cells.

### Lhx6 affected the functions of HEPM cells by regulating mitophagy

**Fig. 5:**
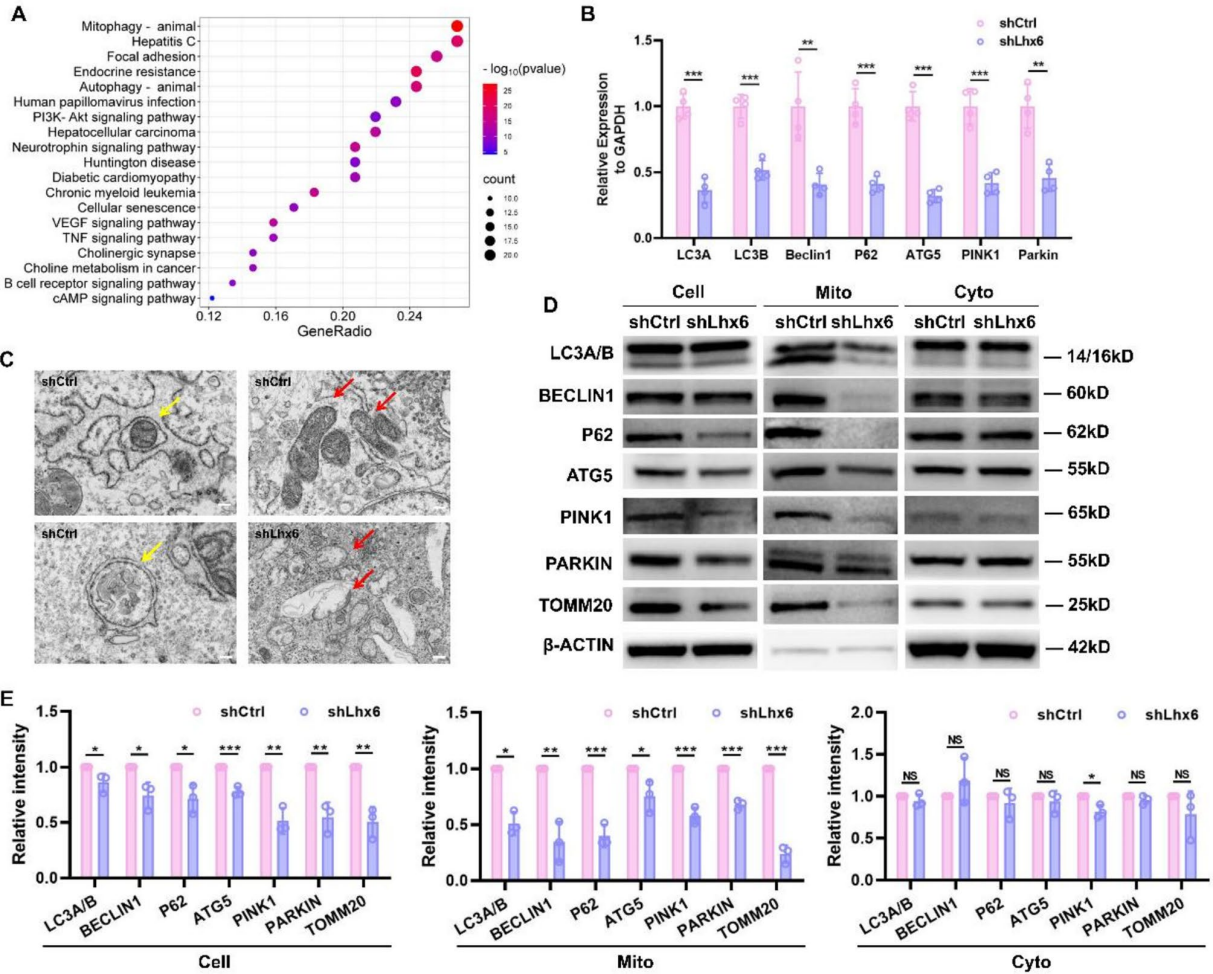
Lhx6 mainly affected the functions of HEPM cells by regulating mitophagy. (**A**) Enriched KEGG pathway analysis of mitochondria-related genes in the shCtrl group and the shLhx6 group. (**B**) qPCR analysis of mitophagy-related genes expression in the shCtrl group and the shLhx6 group. (**C**) Representative transmission electron micrographs (TEM) displaying the morphology of mitochondria in the two groups and mitophagosomes in the shCtrl group. Red arrows point to the mitochondria and the yellow arrows point to the mitophagosomes. Scale bar = 2 μm. (**D**) Western blots of the mitophagy-related markers (LC3A/B, BECLIN1, P62, ATG5, PINK1, PARKIN, TOMM20) of HEPM cells, mitochondria and cytoplasm in the shCtrl group and the shLhx6 group. (**E**) Densitometric analyses of blots showing the protein values of LC3A/B, BECLIN1, P62, ATG5, PINK1, PARKIN and TOMM20 of HEPM cells, mitochondria and cytoplasm in the shCtrl group and the shLhx6 group. Data in the bar diagrams are represented as mean ± SD. N = at least 3 biological replicates. Circles correspond to each tested individual. ^NS^*P >* 0.05, *P < 0.05, **P < 0.01, ***P < 0.001

The critical regulatory role of Lhx6 in RA affecting mitochondrial and cell proliferation and migration functions had been verified. Lhx6’s potential role in regulating mitochondrial function was then explored in detail. After sequencing the mRNA transcriptome of the shCtrl and shLhx6 groups, we analyzed the KEGG-enriched pathway for mitochondria-related genes (Fig. 5A). Mitophagy-related genes enriched most in the analysis, which prompted us to conduct further investigation. Consequently, we examined the mRNA levels of mitophagy-related genes in cells of shCtrl and shLhx6 groups (Fig. 5B). and the results showed that the levels of mitophagy markers including LC3A, LC3B, Beclin1, P62, ATG5, PINK1, and Parkin were decreased in the shLhx6 group, suggesting that knockdown of Lhx6 inhibited mitophagy. The HEPM cells were examined directly with transmission electron microscopy to observe mitochondrial changes (Fig. 5C). More autophagosomes were observed wrapping around mitochondrial fragments in the shCtrl group, which implied the normal occurrence of the mitophagy process. In addition, healthy mitochondrial morphology with clearly visible mitochondrial cristae could be observed. On the contrary, in the shLhx6 group, it was hard to observe mitochondrial autophagosomes and the mitochondrial morphology appeared to be swollen and vacuolated, with mitochondrial cristae missing. Moreover, we quantified the protein expression levels of mitophagy markers (Fig. 5D&E). We first extracted cellular proteins for detection, and the levels of protein expression of mitophagy markers (LC3A/B II, Beclin1, P62, ATG5, PINK1, Parkin) were all declined to various extent in shLhx6 group. To clarify the changes at the mitochondrial level, we next extracted cellular mitochondria and cytoplasm and performed Western blots separately, the result of which demonstrated that the mitochondria in the shLhx6 group exhibited a more significant down-regulation of the mitophagy markers, whereas differences in marker levels in the cytoplasm were statistically insignificant compared to those in the Ctrl group. It had been demonstrated, therefore, that Lhx6 knockdown inhibited cellular mitophagy.

### RA-induced inhibition of HEPM mitophagy could be corrected by overexpression of Lhx6

**Fig. 6:**
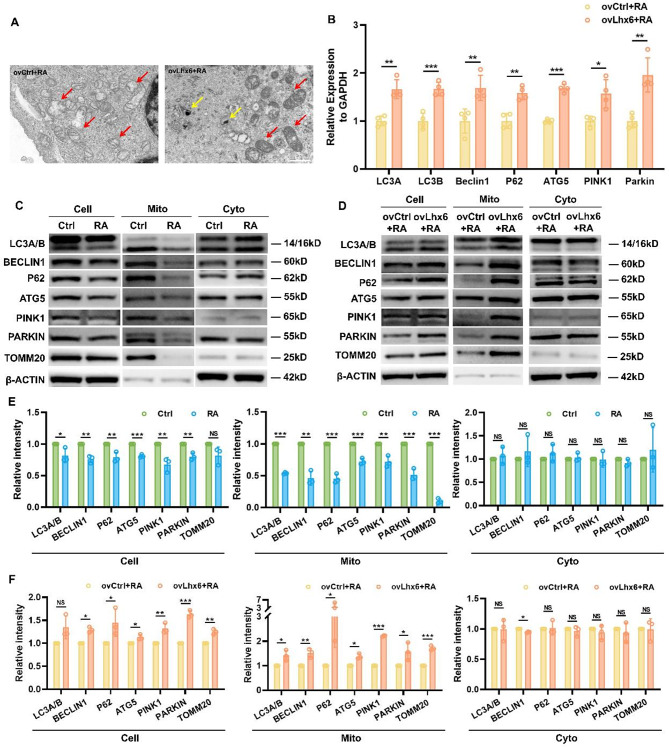
RA-induced inhibition of HEPM mitophagy could be corrected by overexpression of Lhx6. (**A**) Representative transmission electron micrographs (TEM) displaying the morphology of mitochondria in the two groups and mitophagosomes in the ovLhx6 + RA group. Red arrows point to the mitochondria and the yellow arrows point to the mitophagosomes. Scale bar = 10 μm. (**B**) qPCR analysis of mitophagy-related genes expression in the ovCtrl + RA group and the ovLhx6 + RA group. Western blots showing the mitophagy-related markers (LC3A/B, BECLIN1, P62, ATG5, PINK1, PARKIN, TOMM20) of HEPM cells, mitochondria and cytoplasm in (**C**) the Ctrl group and the RA group as well as (**D**) the ovCtrl + RA group and the ovLhx6 + RA group. Densitometric analyses of blots showing the protein values of LC3A/B, BECLIN1, P62, ATG5, PINK1, PARKIN and TOMM20 of HEPM cells, mitochondria and cytoplasm in (**E**) the Ctrl group and the RA group as well as (**F**) the ovCtrl + RA group and the ovLhx6 + RA group. Data in the bar diagrams are represented as mean ± SD. N = at least 3 biological replicates. Circles correspond to each tested individual. ^NS^*P >* 0.05, *P < 0.05, **P < 0.01, ***P < 0.001

Having discovered the role of Lhx6 in regulating mitophagy, we then explored the effects of overexpression of Lhx6 on RA-induced mitophagy in HEPM cells. By using transmission electron microscopy, we observed that vacuolated mitochondria were reduced and that more mitochondria had intact morphology and mitochondrial cristae in the ovLhx6 + RA group than in the ovCtrl + RA group (Fig. 6A). The transcript levels of mitophagy marker genes in both groups were examined by qPCR and Lhx6 was able to reverse the RA-induced decrease in the transcript levels of mitophagy markers including LC3A, LC3B, Beclin1, P62, ATG5, PINK1, and Parkin (Fig. 6B). In addition, we confirmed that RA treatment inhibits mitophagy by Western blots and quantitative analysis of decreased protein levels of mitophagy markers (Fig. 6C&E). And we further verified that overexpression of Lhx6 reversed the reduction of mitophagy markers at the protein level induced by RA, as shown by a significant increase in the protein levels of LC3A, LC3B, Beclin1, P62, ATG5, PINK1, Parkin (Fig. 6D&F). It was concluded that overexpression of Lhx6 corrected RA-induced inhibition of mitophagy in HEPM cells.

### Abnormal mitophagy inhibited the proliferation and migration of HEPM cells via MAPK signaling pathway

**Fig. 7:**
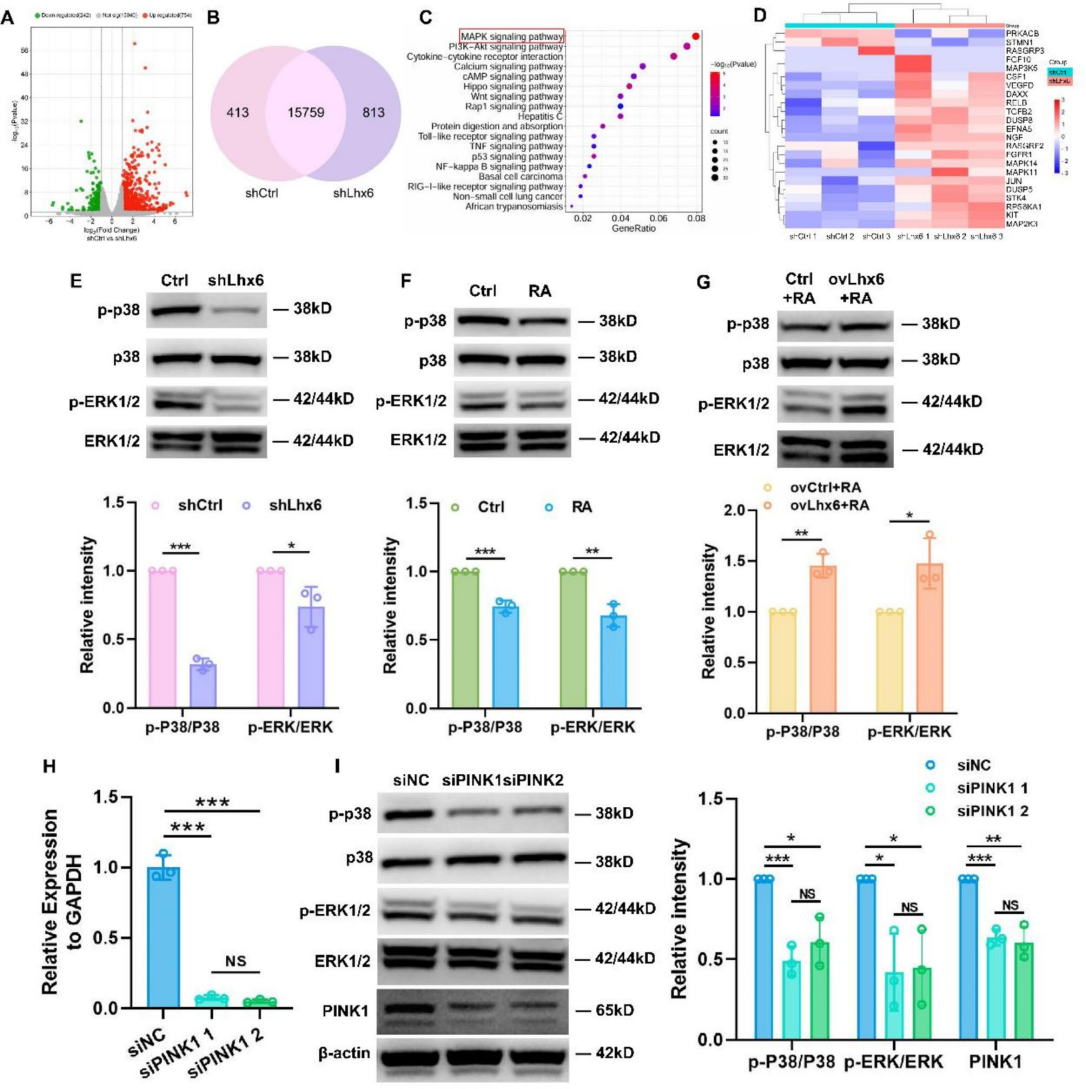
Abnormal mitophagy inhibited the proliferation and migration of HEPM cells by regulating MAPK signaling pathway. (**A**) A volcanic plot representing significantly altered gene numbers in the shCtrl vs. shLhx6 group. (**B**) A Venn diagram showing the distribution of genes in the shCtrl vs. shLhx6 group. (**C**) Enriched KEGG pathway analysis between the shCtrl and shLhx6 group. (**D**) Column-clustered heat map of MAPK signaling pathway-related genes that are differentially expressed between the two groups. The color intensity represents the relative mRNA level; red and blue indicate high and low expression, respectively. (**E**, **F**, **G**) Representative western blots and densitometric analyses showing the p38 and ERK1/2 MAPK signal pathway proteins in each group. (**H**) qPCR analysis to confirm the efficiency of PINK1 knockdown. (**I**) Representative western blots and densitometric analyses showing the p38 and ERK1/2 MAPK signal pathway proteins in each group. Data in the bar diagrams are represented as mean ± SD. N = at least 3 biological replicates. Circles correspond to each tested individual. ^NS^*P >* 0.05, *P < 0.05, **P < 0.01, ***P < 0.001

Further studies were then conducted to determine if Lhx6 was related to changes in cell-associated signaling pathways following mitophagy. This time, the transcriptome sequencing of shCtrl vs. shLhx6 was analyzed again. The volcano plot revealed that the shLhx6 group had both up-regulated genes and down-regulated genes (Fig. 7A), and a Venn diagram showed how genes were distributed in the two groups of cells (Fig. 7B). An enriched KEGG analysis revealed the most obvious differences to be associated with the MAPK signaling pathway (Fig. 7C), while the gene heatmap demonstrated the presence of differences in the expression of genes related to this pathway (Fig. 7D). Transcriptome sequencing results of ovCtrl group vs. ovLhx6 group further confirmed that Lhx6 altered gene expression and signaling pathways in HEPM cells, with significant differences in the MAPK signaling pathway (Fig. S3).

On the basis of the sequencing results, we performed western blots to identify MAPK signaling pathway factors. Both RA induction and knockdown of Lhx6 inhibited p38 and ERK1/2 phosphorylation, indicating that the MAPK signaling pathway was inhibited (Fig. 7E&F). According to Fig. 7G, overexpression of Lhx6 significantly increased phosphorylation of p38 and ERK1/2, despite the inhibitory effects of RA, indicating that the MAPK pathway was activated, and the phosphorylated forms of proteins and the ratio based on the density of bands in each group further confirmed the observations.

Finally, PINK1 was knocked down in HEPM cells by transfection of siRNA (Fig. 7H) to observe if the MAPK signaling pathway was altered. As demonstrated by Western blots and quantitative analysis (Fig. 7I), PINK1 knockdown reduced the p38 and ERK1/2 phosphorylation, suggesting PINK1 inhibition of MAPK pathway activation. As shown by the above experiments, RA interfered with PINK1/Parkin-mediated mitophagy by reducing Lhx6 expression, which in turn regulated MAPK signaling pathway, leading to abnormal proliferation and migration of HEPM cells as a result.

## Discussion

In this study, we explored the role of Lhx6 in RA-induced cleft palate by a series of in vivo and in vitro experiments, and elucidated for the first time that Lhx6 affects the proliferative and migratory function of mesenchymal cells by regulating mitochondrial function, including increased rod-shaped mitochondria, decreased mtDNA copy number and ATP levels, and increased mtROS levels.

The palatal shelf is composed of a mesenchymal core that is epithelially covered and grows from both sides of the tongue vertically, which is then elevated horizontally above the dorsal surface of the tongue along with the enlargement of the jaw and lowering of the tongue. The medial margin epithelium of the horizontal palatal shelf then contacts, adheres, and fuses along its midline, creating a midline epithelial suture (Atukorala and Ratnayake 2021; Hu et al. 2013). The development of the palate mainly involves proliferation, migration, osteogenesis, and epithelial-mesenchymal transition, and problems at any point in this complex process can be the cause of cleft palate (CP). Multiple genes are involved in regulating this process and have been studied from various angles to uncover the mechanisms of EPM alteration during cleft palate development. The researchers found that knockdown of Cxcr4 inhibited EPM cell migration similarly to plerixafor, which inhibited cell migration in the same manner. Supplementation with C-X-C motif chemokine ligand 12 (CXCL 12) partially reversed the inhibition of cell migration in Cxcr4 knockouts. (Zheng et al. 2023). RA is widely used in the craniomaxillofacial malformations, which is explored by our group before (Wang et al. 2019b; Wang et al. 2017). Dong et al. suggested that RA inhibited the expression of RBP4, which led to a decrease in the expression of cell cycle protein D1 and an increase in the expression of p27, causing growth inhibition of EPM cells. Overexpression of RBP4 reversed the inhibitory effect of atRA and promoted cell proliferation through the ERK1/2 and AKT signaling pathway (Dong et al. 2017). In addition, microRNA alterations in EPM cells during cleft palate have also been reported in RA-induced cleft palate in mice (Yan et al. 2022; Yoshioka et al. 2022). These studies suggest that RA-induced cleft palate is inevitably accompanied by abnormal migration and proliferation of embryonic palatal mesenchymal stromal cells through complex gene regulation. Additionally, we here showed that RA caused cleft palate by inhibiting Lhx6 expression.

Homeobox genes have been conducted around the regulation of MSC proliferation and migration, such as spatiotemporal regulation of cell fate in tooth development and regeneration by the Lhx6/8 (Huang et al. 2021; Wang et al. 2019a; Zhou et al. 2015b). The importance of Lhx6/8 in palatal development has also been recognized, but the role of Lhx6 in cleft palate and its specific mechanisms have not been studied in depth by our group, and only a small number of studies have reported the role of the Lhx6 gene in cleft palate development. In the embryo, Lhx6 regulates cell proliferation in the cervical ring and promotes cell differentiation in the anterior region of the incisors for normal craniofacial and dental development (Zhang et al. 2013), and Lhx6 can also synergize with other genes in the embryo to determine developmental diversity and regulate cellular function and fate (Dvoretskova et al. 2024). Cesario et al. reported on the molecular genetic pathways midstream and downstream of Lhx6/8 and found that Lhx6/8 negatively regulates p57^Kip2^ expression in the expected palatal region to allow for a sufficient level of cell proliferation to promote normal palatal development (Cesario et al. 2015). They further revealed positively regulated target genes and possible enhancers of Lhx6/Lhx8 in the first pharyngeal arch (PA1), uncovering new links between Lhx and other important regulators of craniofacial development (Cesario et al. 2022). Moreover, genetic intervention targeting Lhx6 can regulate early embryonic development and its informational crosstalk with the maternal uterus (Shi et al. 2021), and knockdown of Lhx6 leads to neurophysiological and behavioral abnormalities during development (Elam et al. 2024). In addition, decreased expression of Lhx6 has been reported in diabetes, tumors, and inflammatory environments, and Lhx6 is also involved in signaling pathways that regulate disease progression and regression, including p38, NF-κB and Wnt/β-catenin (Chen et al. 2019; Hwang et al. 2024). Consistently, we here showed that RA caused cleft plate and mitochondrial dysfunction by inhibiting Lhx6 expression, and that intervention in the Lhx6 levels could restore mitochondrial function.

Mitophagy is a selective form of macroautophagy used by cells to degrade mitochondria. Ubiquitin-mediated mitophagy includes PTEN-induced putative kinase 1 (PINK 1)-dependent and Parkin-dependent mitophagy pathways, which promote mitochondrial degradation in a ubiquitin-dependent manner, and comprises the three components of the mitochondrial damage sensor (PINK 1), the signaling amplifier (Parkin), and the signaling effector (ubiquitin chain) (Li et al. 2023). Mitophagy-mediated mitochondrial clearance plays an important role in many processes, including early embryonic development, cell proliferation, differentiation, inflammation and apoptosis (Onishi et al. 2021). HOXD 9 targeting the HRD 1 promoter region and regulating downstream PINK1-mediated mitophagy significantly enhances the invasive capacity of endothelial progenitor cells (EPCs). Although the role of PINK1/Parkin-mediated mitophagy in the activity of various cells has been reported, its regulatory effect on mitochondria in HEPM cells was found for the first time in this study. Our results showed that PINK1/Parkin-mediated mitophagy was inhibited in RA-treated HEPM cells and could be reversed by overexpression of Lhx6, which was regulated at both transcriptional and translational levels in PINK1/Parkin and other mitophagy genes, with more significant differences in experimental results at the mitochondrial level, suggesting its ability to activate RA-induced mitophagy in HEPM cells.

**Fig. 8:**
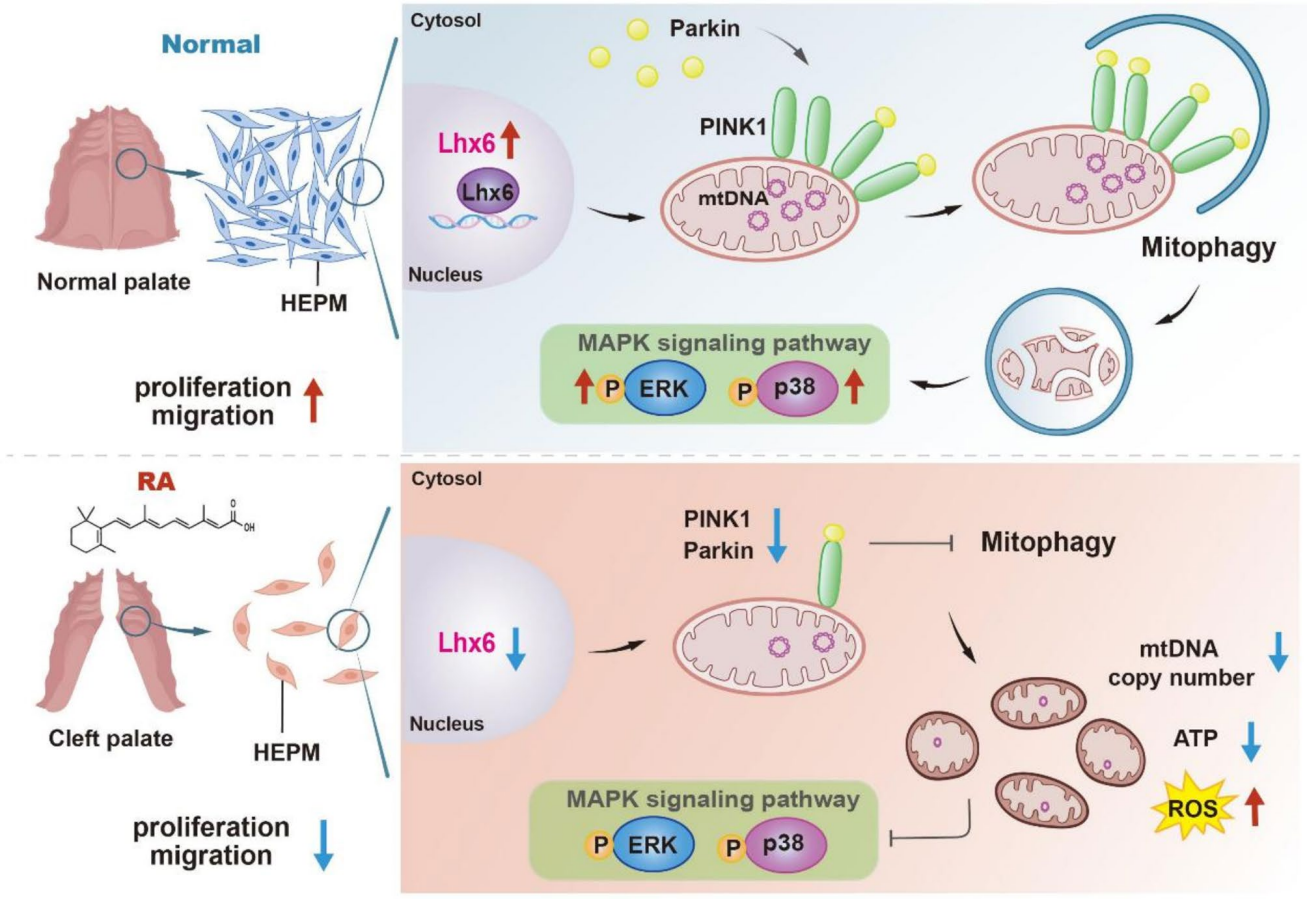
Schematic illustration of the mechanism by which RA leads to HEPM cells dysfunction

Additionally, we found that p38 and ERK1/2 MAPK signaling pathways are downstream pathways of PINK1/Parkin. Apart from the MAPK signaling pathway, there are multiple signaling pathways involved in RA-induced cleft palate. Early in palatal development, retinoic acid completely inhibits the Wnt/β-catenin signaling pathway through binding and activation of retinoic acid receptor (RAR), leading to retinoic acid-induced cleft palate, and the PI3K/Akt signaling pathway is also involved in RA (Hu et al. 2013). Moreover, Shi et al. found that PTEN/AKT/mTOR autophagy signaling could maintain the stem cell properties of EPM cells and prevent the formation and development of cleft palate (Shi et al. 2019). In this study, we recreated a realistic scenario to assess the impact of RA on embryonic palatal development using a widely accepted RA-induced animal model, subsequently delving into the molecular mechanisms underlying RA’s effects on HEPM cells. However, our research did not include in vivo validation of gene-targeted interventions. For future research, it would be beneficial to utilize conditional knockout transgenic mice or conduct in vivo DNA transfections to broaden our understanding of the regulation network of Lhx6 in craniomaxillofacial development and further explore its contribution to embryonic resistance to RA. In conclusion, the action of RA on EPM cells leading to cleft palate is an extremely complex process, and the present study, building upon the team’s previous research achievements and focusing on mechanism exploration, further revealed the biological process of Lhx6 in promoting the proliferation and migration of HEPM cells by mediating PINK1/Parkin mitophagy, at least partially via activating the MAPK signaling pathway, as shown in Fig. 8. This research pioneers the discovery of Lhx6’s role in mediating mitophagy associated with the development of cleft palate, offering novel insights into the etiology of malformations and highlighting the application value of molecular research in the screening of developmental malformations. As a promising molecular marker, the deficiency of Lhx6 could potentially elevate the risk of cleft palate occurrence, and genetic screening for Lhx6 and its related biomarkers may play a contributive role in the prevention of such malformations.

## Conclusion

In conclusion, RA has been found to induce dysfunction in HEPM cell proliferation and migration, leading to the development of cleft palate through the downregulation of Lhx6 and subsequent mitochondrial dysfunction. Mechanistically, Lhx6 is essential for mitochondrial function maintenance via the PINK1/Parkin-mediated mitophagy as well as the p38 and ERK1/2 MAPK signaling pathways. Our research underscores the significance of Lhx6 in developmental processes and proposes the restoration of Lhx6 as a potential therapeutic strategy for the prevention of cleft palates.

## Electronic supplementary material

Below is the link to the electronic supplementary material.

Supplementary Material 1

## Data Availability

No datasets were generated or analysed during the current study.

## Abbreviations

ATP: Adenosine Triphosphate
ATG5: Autophagy 5
atRA: All-Trans Retinoic Acid
CCK-8: Cell Counting Kit-8
CLP: Clefts of the lip and/or palate
CP: Cleft Palate
CXCL 12: C-X-C motif chemokine ligand 12
DEGs: Differentially Expressed Genes
EPM: Embryonic Palatal Mesenchymal
EPCs: Endothelial Progenitor Cells
ERK1/2: Extracellular Regulated Protein Kinases
FBS: Fetal Bovine Serum
GAPDH: Glyceraldehyde-3-Phosphate Dehydrogenase
HEPM: Human Embryonic Palatal Mesenchymal
H&E/HE: Hematoxylin and eosin
ICT1: Immature Colon Carcinoma Transcript 1
JC-1: 5,5’,6,6’-tetrachloro-1,1’,3,3’-tetraethylbenzimidazolcarbocyanine iodide
KEGG: Kyoto Encyclopedia of Genes and Genomes
LC3: Light chain 3
Lhx6: LIM Homeobox6
Lhx8: LIM Homeobox8
MAPK: Mitogen-Activated Protein Kinase
MSC: Mesenchymal stem cell
MT ND1: NADH Dehydrogenase 1 (mitochondrion)
mtDNA: Mitochondrial DNA
mtROS: Mitochondrial Reactive Oxygen Species
mtRQC: Mitoribosome-Associated Quality Control
OXPHOS: Oxidative Phosphorylation
PCR: Polymerase Chain Reaction
PINK1: PTEN induced putative kinase 1
PTEN: Phosphatase and Tensin Homolog
p62: Prostacyclin 62
p38: Prostacyclin 38
qPCR: Quantitative Real-Time PCR
RA: Retinoic acid
RAR: Retinoic Acid Receptor
RLU: Relative Light Unit
ROS: Reactive Oxygen Species
SD: Standard Deviation
siRNA: Small Interfering RNA
TEM: Transmission Electron Micrographs
TOMM20: Translocase of Outer Mitochondrial Membrane 20
α-MEM: α-Minimum Essential Medium
