# Supplementary material for: Lhx6 deficiency causes human embryonic palatal mesenchymal cell mitophagy dysfunction in cleft palate
Source: Mol Med. 2024 Oct 22;30:183. doi: 10.1186/s10020-024-00960-2 (PMC11494960; doi:10.1186/s10020-024-00960-2)
Supplement: Supplementary file 1 — Supplementary Material 1 [file 10020_2024_960_MOESM1_ESM.docx]

**Supplementary material**

**Table S1：**


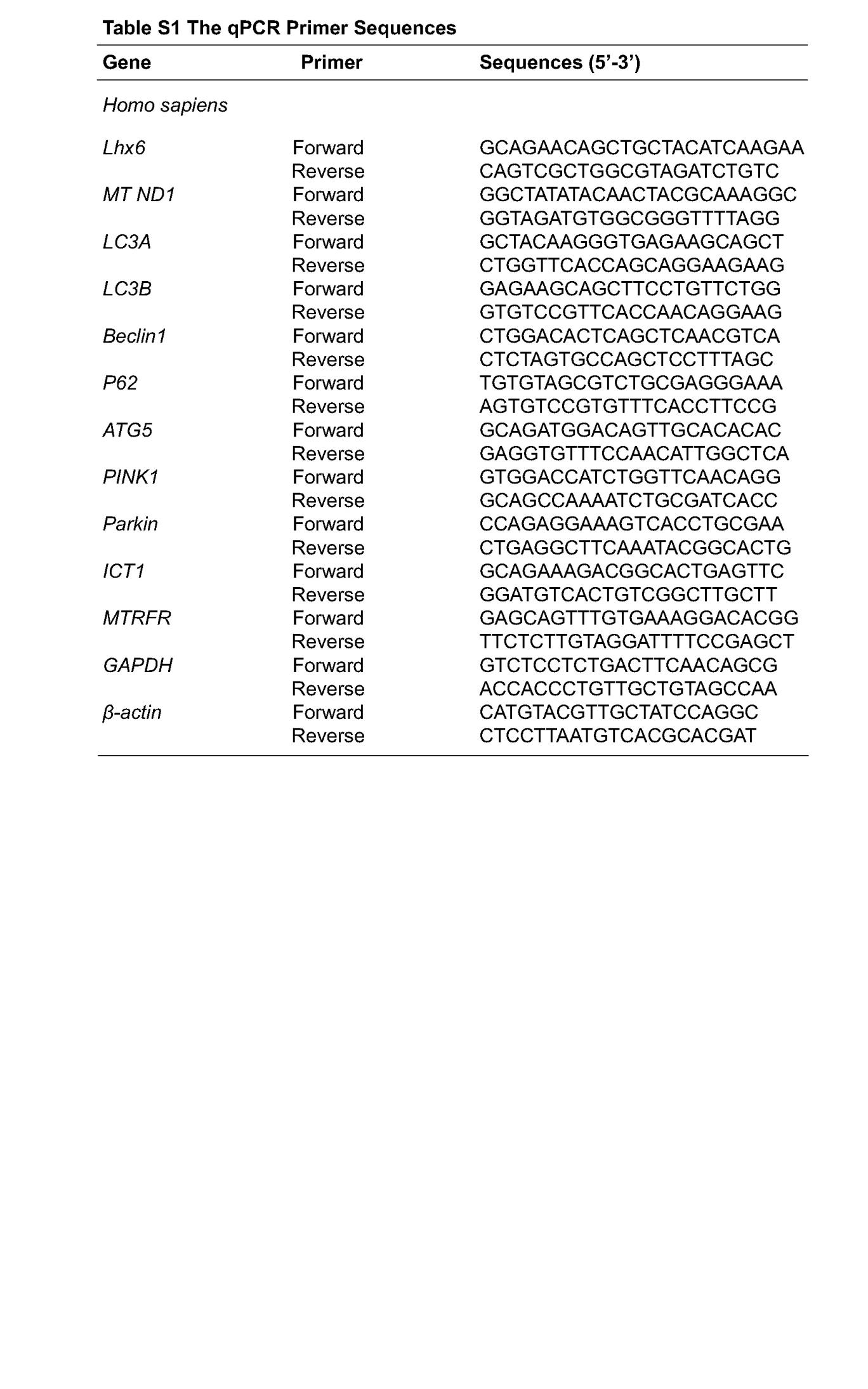


Table S1 The qPCR Primer Sequences.

**Fig. S1:**


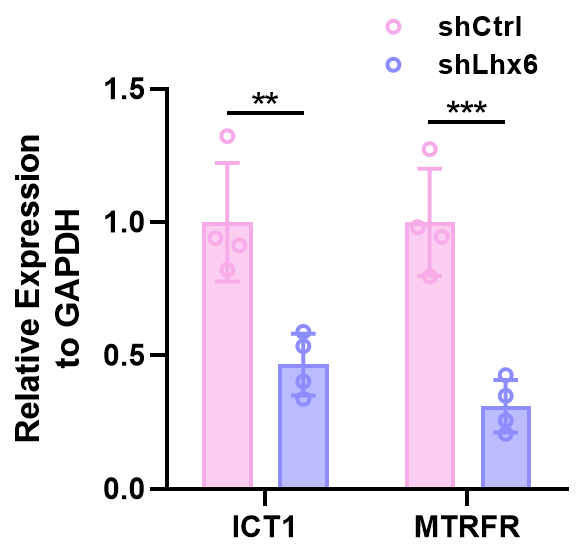


Fig. S1 qPCR analysis of mtRQC-related genes expression in the shCtrl group and the shLhx6 group.

**Fig. S2:**


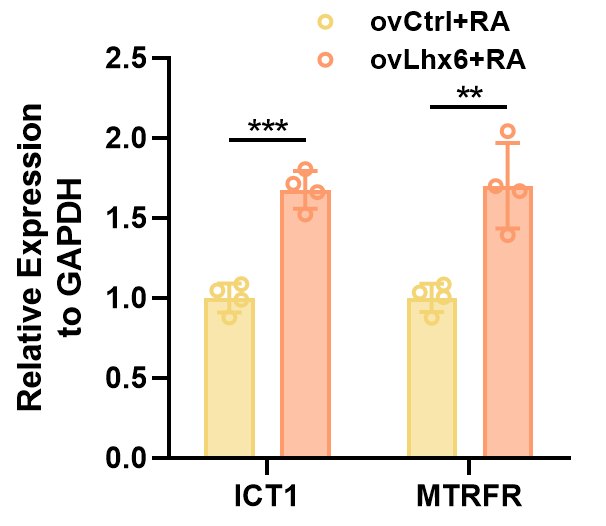


Fig. S2 qPCR analysis of mtRQC-related genes expression in the ovCtrl+RA group and the ovLhx6+RA group.

**Fig. S3：**


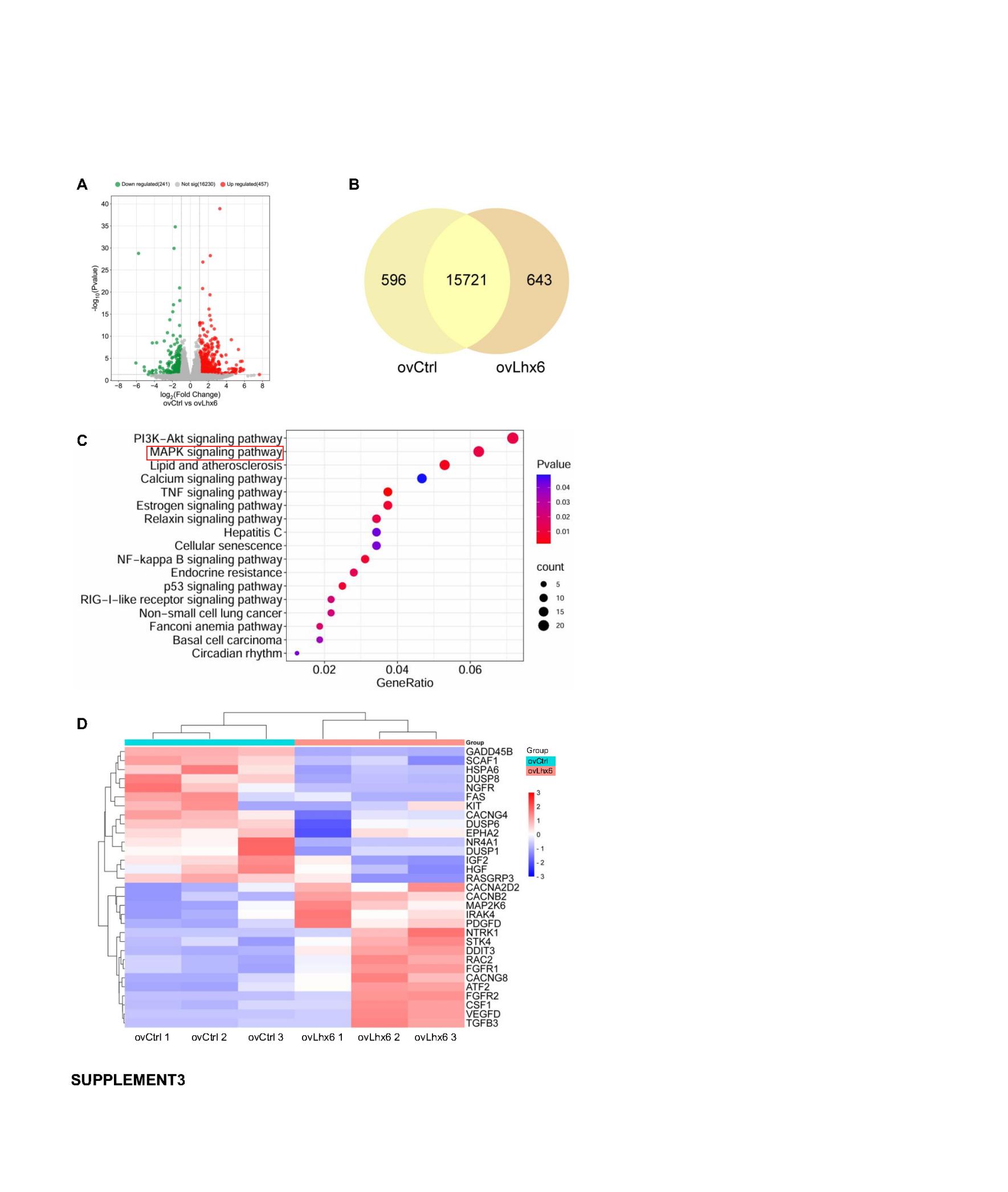


Fig. S3 (A) A volcanic plot representing significantly altered gene numbers in the ovCtrl vs ovLhx6 group. (B) A Venn diagram showing the distribution of genes in the ovCtrl vs ovLhx6 group. (C) Enriched KEGG pathway analysis between the ovCtrl vs ovLhx6 group. (D) Column-clustered heat map of MAPK signaling pathway-related genes that are differentially expressed between the two groups. The color intensity represents the relative mRNA level; red and blue indicate high and low expression, respectively.
